# Green Synthesis of Bioplastics from Microalgae: A State-of-the-Art Review

**DOI:** 10.3390/polym16101322

**Published:** 2024-05-08

**Authors:** Adegoke Isiaka Adetunji, Mariana Erasmus

**Affiliations:** Centre for Mineral Biogeochemistry, University of the Free State, Bloemfontein 9301, South Africa

**Keywords:** bioplastics, production, microalgae, circular bioeconomy, biopolymers, applications

## Abstract

The synthesis of conventional plastics has increased tremendously in the last decades due to rapid industrialization, population growth, and advancement in the use of modern technologies. However, overuse of these fossil fuel-based plastics has resulted in serious environmental and health hazards by causing pollution, global warming, etc. Therefore, the use of microalgae as a feedstock is a promising, green, and sustainable approach for the production of biobased plastics. Various biopolymers, such as polyhydroxybutyrate, polyurethane, polylactic acid, cellulose-based polymers, starch-based polymers, and protein-based polymers, can be produced from different strains of microalgae under varying culture conditions. Different techniques, including genetic engineering, metabolic engineering, the use of photobioreactors, response surface methodology, and artificial intelligence, are used to alter and improve microalgae stocks for the commercial synthesis of bioplastics at lower costs. In comparison to conventional plastics, these biobased plastics are biodegradable, biocompatible, recyclable, non-toxic, eco-friendly, and sustainable, with robust mechanical and thermoplastic properties. In addition, the bioplastics are suitable for a plethora of applications in the agriculture, construction, healthcare, electrical and electronics, and packaging industries. Thus, this review focuses on techniques for the production of biopolymers and bioplastics from microalgae. In addition, it discusses innovative and efficient strategies for large-scale bioplastic production while also providing insights into the life cycle assessment, end-of-life, and applications of bioplastics. Furthermore, some challenges affecting industrial scale bioplastics production and recommendations for future research are provided.

## 1. Introduction

Plastics are produced from oil, natural gas, coal, or petrochemicals. These carbon-based polymers have transformed our lives in diverse ways by opening avenues for vital developments in many industries. In recent years, plastic production has increased tremendously owing to rapid population growth and advancements in the use of technologies [1]. Worldwide plastic production is expected to reach 445 million tons, with an additional increase to 589 million tons, by 2050 [1]. These synthetic materials are stable, transparent, lightweight, versatile, durable, affordable, and resistant to corrosion, with high strength [2,3]. However, despite their immense benefits, overuse of these fossil-based polymers results in serious impacts on the environment, causing pollution, global warming, and fossil fuel depletion, due to their hydrophobicity and huge resistance to biodegradation [4,5]. In addition, synthetic plastics are recalcitrant in nature and release toxic chemicals to the environment, especially when indiscriminately disposed of, thereby polluting water bodies, and adversely affecting ecosystems [6,7,8]. To overcome the abovementioned challenges, there is a need to produce plastics from natural renewable biomass sources.

**Figure 1:**
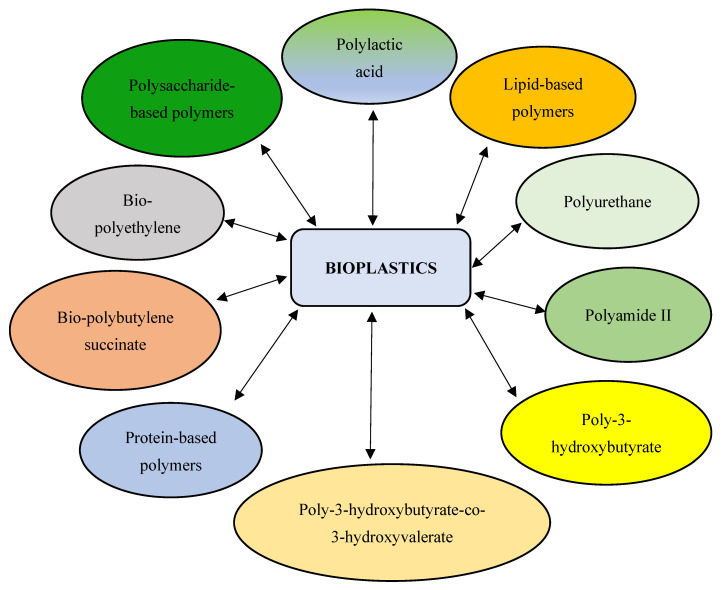
Schematic diagram depicting different microalgae-derived polymers.

Bioplastics are degradable or non-degradable biobased polymers [9,10,11]. They are produced from natural polymers of plant, animal, or microbial origin. Microorganisms serve as an excellent source for bioplastics production due to their ease of cultivation, rapid growth rate, high productivity, ease of genetic manipulation, etc. [12]. The use of microalgae as a feedstock for bioplastic production is highly preferred, owing to the ability of these photoautotrophic organisms to grow at a faster rate with high biomass. Unlike plant-based bioplastics, the use of microalgae does not lead to food competition for human consumption [13]. In addition, microalgae have fewer nutritional demands and thrive well in non-arable environments (e.g., wastewater) [14]. Microalgae consume inorganic compounds for growth and production of certain metabolites (e.g., proteins, carbohydrates, and lipids). These metabolites are utilized for various applications, including the synthesis of polysaccharides (such as alginate, carrageenan, and agar) for bioplastics production [15,16]. In other words, microalgae serve as a sustainable source for the commercial production of biopolymers via cultivation or natural harvest [8]. Bioplastics are produced by conversion of algal biomass through fermentation, plasticization, blending, and compatibilization processes [17]. According to a recent survey by European Bioplastics, global bioplastics production is predicted to increase from 2.4 million tons in 2022 to 7.5 million tons by 2026 as a substitute for conventional plastics [18]. Microalgae-derived plastics are economical, highly recyclable, biocompatible, biodegradable, energy efficient, flexible, have a lesser carbon footprint, and generate no toxic by-products, leading to a more sustainable circular economy [13]. However, bioplastics are brittle with low melt strength and weak barrier properties. These include bio-polybutylene succinate (bio-PBS), polylactic acid (PLA), polyhydroxybutyrate (PHB), polyurethane (PU), bio-polyethylene (bio-PE), polyhydroxyalkanoates (PHAs), and starch-based, cellulose-based, lipid-based, and protein-based biopolymers [19] (Figure 1). These biobased plastics are employed in a variety of industrial, agricultural, and biomedical applications [20,21,22,23,24]. The present review provides insights into techniques used by microalgae for the synthesis of biopolymers and bioplastics, while also elucidating strategies for the optimization of microalgae-derived bioplastics for potential applications in industries, biomedicine, and agriculture.

## 2. Sources of Bioplastics

### 2.1. Plants

Natural biopolymers of plant origin have great potential to be employed as feedstocks for bioplastics production [25]. These include corn starch, wheat starch, cassava, sawdust, sugarcane bagasse, vegetable fats and oils, etc. The lipids or sugars of these biopolymers are subjected to fermentation or chemically modified to produce bioplastics [6]. In addition, natural polysaccharides, such as gluten and cellulose, can also be transformed into bioplastics [25]. The supplementation of sugarcane bagasse with starchy materials has been shown to enhance the tensile and mechanical properties of bioplastics [26]. Coconut husk fibers can be employed as a support material in bioplastics produced from cassava starch [27]. The modification of cassava starch with glycerol, vinegar, and water has been shown to result in the formation of bioplastic sheets [28]. The use of plant cellulose as a biobased feedstock in the synthesis of bioplastics has been reported [29,30,31]. Studies on the synthesis of lipid-based bioplastics using palm oil, soya bean oil, olive oil, linseed oil, and castor oil have also been reported [32]. Plant oil-based bioplastics are thermostable, with better tensile strength and elongation [33].

### 2.2. Animals

Animal by-products, such as hides, skins, and tallow, from tannery industries are used as a sustainable and economical source for the synthesis of biodegradable bioplastics [34]. Furthermore, animal proteins, including collagen, gelatin, and keratin, are suitable for compostable bioplastic production, due to their favorable functional properties [35]. Myofibrillar proteins from fish co-products have been demonstrated to produce plastic films with high transparency, low water vapor permeability, and exceptional mechanical properties, making them suitable for the packaging of food products [36]. Chitin is a major constituent of the exoskeleton of arthropods (e.g., shrimps, crabs, and crustaceans). The removal of the acetyl group from chitin results in the formation of chitosan. Biodegradable plastic films fabricated from chitosan have been shown to demonstrate robust mechanical, transparency, and antimicrobial properties [35]. In addition, Alvarez-Castillo et al. [37] employed porcine plasma protein for the synthesis of superabsorbent composite bioplastic.

### 2.3. Microorganisms

A variety of microorganisms (including bacteria, fungi, yeasts, and microalgae) produce and store bioplastics (e.g., PHAs) as carbon and energy sources. The extent of production of the polymers is determined by the microbial and substrate type, the physiology of the organisms, as well as the availability of appropriate nutritional and physicochemical parameters [38]. Bacteria such as *Citrobacter*, *Pseudomonas*, *Enterobacter*, *Klebsiella*, *Rhizobium*, *Azotobacter*, and *Alcaligenes* have been reported as being viable for bioplastic production [39,40,41]. These microbes can survive in the presence or absence of ample nutrients for PHA synthesis [38]. For instance, Mozejko-Ciesielska et al. [42] reported a highest yield of 0.42 g/L PHA from *Halomonas alkaliantarctica* using dairy waste as a carbon source. Similarly, *Bacillus megaterium* was found to secrete 0.98 g PHA/carbon in the presence of cacao fruits residue [43]. In addition, some bacteria, and yeasts (such as *Saccharomyces cerevisiae*, *Candida krusei*, *Rhodotorula glutinis*, *Ralstonia eutropha*, and *Kluyveromyces africans*) that utilize polyphosphate complexes in their membrane transport produce low-molecular weight PHB [44,45]. Drakonaki et al. [46] reported 310 µg PHB production by *Pseudomonas* sp. phDV1 after 72 h in the presence of phenol per gram of cells. However, the use of microalgae is a promising, sustainable, and cost-effective bio-factory for bioplastics production. This can be achieved by the direct use of algal biomass, or by blending with other materials [19]. Microalgae such as *Spirulina* sp., *Enteromorpha crinite*, *Chlorella* sp., *Laminaria japonica*, and *Ulva armoricana* have been reported as suitable for bioplastic synthesis [47,48,49,50,51]. Cyanobacteria, including *Muscorum* sp., *Synechococcus* sp. and *Synechocystis* sp., have been shown to produce 30–80% PHB [52,53,54]. Similarly, PHA synthesis by *Calothrix scytonemicola* has been reported [55]. Other microalgae well known for bioplastics production include *Chlorogloea fritschii*, *Scenedesmus almeriensis*, *Neochloris oleoabundans*, *Phaeodactylum tricornutum*, and *Nannocloropsis gaditana* [55,56,57,58,59]. However, *Chlorella* sp. are the best and most prominent microalgae for the production of biobased bioplastics [19].

## 3. Biopolymers Produced from Microalgae and Their Properties

### 3.1. Polyhydroxyalkanoates

Polyhydroxyalkanoates are polyesters synthesized by cyanobacteria and microalgae, and are often utilized as natural polymers for the production of bioplastics. These polymers have great potential and interesting properties, including biodegradability, biocompatibility, robust plasticizing capacity, and recyclability, making them the most preferred among a wide range of biopolymers [60]. They are water-insoluble and resistant to ultraviolet and hydrolytic attack [41]. Additionally, PHAs possess inherent mechanical and thermoplastic properties akin to conventional petrochemical plastics with tensile strength and Young’s modulus in the range of 18–40 MPa and 0.6–3.8 GPa, respectively [61]. PHAs are categorized into three groups, based on their carbon chain length. These include short-chain PHAs (≤5 carbon atoms), medium-chain PHAs (6 ≤ 14 carbon atoms), and long-chain PHAs (≥15 carbon atoms) in biopolymer backbones [62]. The short-chain length PHAs are the most widely used in food packaging and disposal products [63]. There are about 150 different biodegradable monomers of PHAs that have been identified [64,65]. These include poly(3-hydroxybutyrate) (P(3HB), poly(3-hydroxybutyrate-co-3-hydroxyvalerate) (P3HB-co-3HV), poly (3-hydroxybutyrate-co-4-hydroxybutyrate) (P(3HB-co-4HB), and poly (3-hydroxybutyrate-co-3-hydroxyhexanoate (P3HB-co-3HH) [66]. However, P(3HB) and P(3HB-co-3HV) are the most prominent polymers [67].

PHAs are stored in the inclusion bodies of microalgae as a source of carbon and energy during the stationary phase and contribute to more than 80% of the cell’s weight [68]. Under environmental stress conditions, PHA accumulation in microalgae can be triggered when P and N are inadequate in the culture medium [68,69]. For instance, *Arthrospira platensis* was shown to secrete 5.8 mg PHA/g when deficient in N [70]. Similarly, PHA (29% *w*/*w*) was synthesized by *Scenedesmus* sp. when grown in phosphorus-lacking conditions [68]. The synthesis of these polymers by microalgae occurs in the presence of acetyl coenzyme A during cultivation in a nutrient-deficient medium. In addition, PHAs are usually produced from expensive substrates. However, synthesis from cost-effective renewable feedstock makes these biopolymers attractive targets for the plastic industry [71]. Furthermore, PHAs can be produced by fermentation in the presence of plant-derived sugars and oils as carbon and energy sources [67]. The physical properties of PHAs are influenced by the type of organism, monomer composition, the polymer extraction method used, and organism growth conditions [19]. However, cyanobacteria are the best known PHA producers [72]. PHAs have been reported in the range of 1–25% dry weight in some cyanobacteria [73]. For instance, PHA production was found to be 21%, 25%, 3.3%, 14%, and 7.4% by *Nostoc* sp., *Calothrix* sp., *Synechocystis* sp., *Oscillatoria* sp., and *Spirulina* sp., respectively [73,74,75,76,77,78].

PHBs are short chain homopolymers of hydroxybutyrate, consisting of four carbon atoms in the backbone [13]. Hempel et al. [56] reported PHB levels of approximately 10.6% of algal dry weight in *Phaeodactylum tricornutum*. Fluorescence and electron microscopic analyses revealed the accumulation of these bioplastics in granule-like structures in the cytosol. In addition, *Microcystis aeruginosa* has been shown to exhibit PHB concentrations of up to 0.49 ± 0.5 mg/L [79]. Selvaraj et al. [80] recorded maximum 80% PHB production by *Chlorella* sp. The highest recorded PHB content of 27% dry weight was achieved by Mehariya et al. [81] during cultivation of *Chlorococcum* sp. in BG-11 medium supplemented with sugar-rich hydrolysate (carbon source). Troschl et al. [82] reported an average of 12.5% PHB concentration during cultivation of *Synechocystis* sp. CCALA192 for 75 d in the presence of CO_2_ as sole carbon source. In general, PHA has attracted much attention for use in medicine and various industries, such as in the production of packaging materials, automotive components, home appliances, drug carriers, biodegradable implants, and biocontrol agents [83,84,85,86].

### 3.2. Polylactic Acid

Polylactic acid (PLA) is a low-molecular weight, biodegradable, biobased, and thermoplastic polymer that can be obtained by chemical synthesis or the fermentation of algal biomass (feedstock) to produce monomeric lactic acid [8]. The polymerization of lactic acid results in the formation of PLA for bioplastic production. In addition, PLA can also be synthesized by lactide chain development, or ring-opening [19]. PLA requires a lower amount of feedstock (sugar) and can be copolymerized with other polyesters for enhanced performance in various applications. For instance, PLA has been shown to be useful in packaging applications when strengthened with nanocellulose and microcellulose fibrils [87]. The global market value of PLA is projected to reach USD 5.9 billion by 2027 [88,89]. It exists in three different forms, namely poly (D-lactide), poly (L-lactide), and poly (D,L-lactide). The homopolymers of PLA, consisting of pure L or D-lactic acid monomers, are semicrystalline; the PLA heteropolymers (e.g., D,L-lactic acid) are amorphous in nature [90,91]. In comparison with conventional thermoplastics, PLA is compatible with different fibers, biocompatible, non-toxic, possesses outstanding mechanical strength with easy fabrication, and requires low processing temperatures [92]. In addition, this bioplastic is employed in various industries, including in three-dimensional printing and agriculture, and in the production of prosthetic devices, non-woven binder fibers, bio-sorbents, geotextiles, furniture, and electronic appliances [19,89,93,94].

### 3.3. Polyurethane

Polyurethane (PU) is synthesized by the polycondensation reaction of polyols and isocyanates, which yields a flexible foam product [95]. It consists of urethane groups in its chemical structure and is recognized as a promising polymer with distinct properties, including rigidity, elasticity, and thermoplasticity. The polyols are available in different forms. However, the resources required for their synthesis are largely chemical based. Synthetic PU is expensive, toxic, and non-biodegradable [96]. Therefore, production of PU from renewable resources has attracted the attention of many researchers since this approach promotes waste reduction and sustainability [97,98]. Microalgae can serve as a source for polymer and polyol preparation. For instance, Phung Hai et al. [99] synthesized novel polyols from *Nannochloropsis salina* as a building block for the production of polyurethane foam. In addition, algal oil extraction from *Chlorella vulgaris* and *Enteromorpha* has been shown to result in the formation of biobased thermoplastic PU elastomers [100]. Marlina et al. [101] synthesized algal-based PU film from a casting solution of polyol particles (from *Chaetomorpha linum*) and methylene diphenyl diisocyanate. The epoxidation of commercial crude algal oils has been shown to give rise to the production of polyols and PU [102]. Patil et al. [98] prepared PU coatings from polyols obtained by the chemical transformation of *Chlorella* oil. Polyurethane is employed as a thermoplastic material in medical devices, sealants and elastomers, adhesives, and rigid insulation foams in walls and roofs [95,103]. In addition, this polymer is used in various industries, such as automotive, electronic, construction, textile, and packaging [100].

### 3.4. Cellulose-Based Biopolymers

Cellulose is a water-insoluble polysaccharide consisting of glucose monomers bonded together by β-1,4-glycosidic linkages [104]. It is synthesized by membrane-bound cellulose synthase terminal complexes, which consist of cellulose synthases [105]. It is present in different proportions in the cell wall of microalgae and occurs in varying geometry based on the microalgal taxa [106]. For instance, *Nannochloropsis* sp., *Chorella vulgaris*, and *Kirchneriella lunaris* have been shown to consist of 75% cellulose, 22–25% hemicellulose, and 23% hemicellulose, respectively [107,108,109]. This polymer can attain high levels of polymerization with up to 15,000 glucose subunits. Sugars such as galactose and rhamnose mostly contribute to the hemicellulose fractions of cellulose in some *Chlorella* species [110]. In addition, cellulose possesses distinct hydrophilic and hydrophobic moieties, which confer its stability [111]. Cellulose obtained from microalgae is rigid, biodegradable, crystalline, biocompatible, and fibrous. It is employed as a biobased filler for strengthening in bioplastics synthesis [112]. However, cellulose derived from microalgae is unsuitable for bioplastic production due to its thermal instability, moisture absorption, and non-compatibility with hydrophobic polymers [113]. On the other hand, the incorporation of nanofibers, microcrystalline cellulose, or cellulose nanofibrils from microalgae can act as a reinforcement agent to enhance the biodegradability, mechanical tensile strength, and thermal resistance of bioplastics [112]. For instance, cellulose nanofibrils from *Nannochloropsis oceanica* have been employed as a reinforcing filler [114]. It has been shown that *Cladophora* sp.-derived cellulose can be used to strengthen materials during the synthesis of bioplastics [106]. In one study, cellulose obtained from *Lyngbya* species demonstrated modulus and tensile strength of 24 GPa and 215 MPa, respectively [115]. Cellulose-based bioplastics (e.g., cellulose acetate) are employed in a plethora of applications, including eyeglasses frames, packaging films, three-dimensional printing, electronics, and pharmaceuticals [89,116,117].

### 3.5. Starch-Based Biopolymers

Starch is a polymer that consists of D-glucose subunits connected by glycosidic bonds. In addition, it contains varying amounts of amylopectin (80–90%) and amylose (10–20%) [73]. The higher proportion of amylopectin gives rise to the increased crystallinity of starch, whereas enhanced amylose content results in better tensile strength, lower elongation at break, and higher Young’s modulus, forming fundamental requirements for bioplastics synthesis [118,119]. Starch is stored in microalgae during photosynthesis and stress conditions (such as nutrient deprivation or high light intensity) [120,121]. For instance, Mathiot et al. [122] reported synthesis of substantial amounts (49% w/w) of starch bioplastics under sulfur-scarce conditions by *Chalamydomonas reinhardtii* 11-32A after 20 d. In addition, *Chlorella sorokiniana* produced 38% w/w starch during cultivation at high light intensity and a low nitrogen concentration of 300 µmolm^−2^s^−1^ and 32 mg/L, respectively [121]. The productivity of this polymer is dependent on strain type and growth conditions, and differs significantly among microalgae [123,124]. Remarkably, in one study, *Chlorella* sp. and *Porphyridium marinum* biomass dry weights consisted of 6–13% and 5% starch, respectively [125,126]. In another study, under phototrophic conditions, starch production from microalgae reached about 58 t ha^−1^y^−1^, found to be ten-fold greater than conventional sources (e.g., corn) [127]. Microalgae starch has a small particle size (0.8 to 5.3 µm), making it suitable for use in thin films and flavor carriers [127]. Granular starch is used as a cheap filler in the synthesis of thermoplastics. The combination of thermoplastic starch with biodegradable polymers (e.g., PLA) results in the production of new value-added products with enhanced mechanical and water-resistant properties, as well as lower production costs [73].

### 3.6. Protein-Based Biopolymers

Proteins are macromolecules containing amino acid subunits joined together by amide linkages to form polypeptide chains. The rise in demand for microalgal proteins as an alternative feedstock for bioplastics synthesis has become crucial due to the inappropriateness of plant proteins, given their nature as a well-known food source for human consumption [73]. The protein content of microalgae is determined by several growth environment factors, such as the availability of carbon and nitrogen sources, temperature, light intensity, and quality [128,129,130,131]. The amount of microalgal protein is enhanced when the organism is cultivated under non-stress conditions and in the presence of cheap nitrogen sources (e.g., urea) [73,132,133]. Microalgae-derived proteins are present in huge amounts and can be transformed into bioplastics or thermoplastic blends [134]. For instance, Verdugo and Lim [135] synthesized a novel biobased fiber (200 nm) consisting of 78% protein content from *Botryococcus braunii* using an acidic-electrospinning technique. In addition, microalgal proteins possess better film-forming potentials, adhere to different surfaces, and are biocompatible [136]. The protein structure is influenced by the type of producing microalgal strain. Protein-based bioplastics are employed in various applications, such as in food packaging materials, biomedicine, and biodegradable films [123].

## 4. Production of Bioplastics by Microalgae

Microalgae are an emerging and renewable source for the production of bioplastics [10] (Table 1). These bioplastics can be produced directly with the use of whole microalgal biomass as a base material, or indirectly by fermentation of pre-treated algal biomass [8]. In the former method, all of the components of algal biomass are used for bioplastic synthesis. This approach is cost-effective, recyclable, eco-friendly, reduces carbon footprint and downstream processing costs, and produces clear transparent film [137,138]. In the indirect method, whole algal biomass or spent biomass are fermented for the synthesis of bioplastic precursors. This technique avoids biomass pre-treatment. However, it is time-consuming and results in low yields [8]. In addition, microalgae-based bioplastics are produced by blending algal biomass with other materials, such as starch, cellulose, petrochemical plastics, or bioplastics [139]. This approach prolongs lifespan and enhances the physical and mechanical properties of the bioplastics [139]. For instance, Fabra et al. [140] synthesized thermoplastic films by blending biomass from three microalgal species (*Spirulina*, *Scenedesmus*, and *Nanochloropsis*) with corn starch. Torres et al. [57] prepared bioplastic by mixing biomass (from *Nannochloropsis gaditana*) with polybutylene adipate-co-terephthalate (PBAT). This was found to enhance tensile modulus and reduce elongation at break point. Zhang et al. [141] prepared a polyethylene (PE)-*Chlorella* composite. The *Chlorella* was used as a filler for the thermoplastic PE. Chemical modification was performed on the PE by addition of maleic anhydride to increase the tensile strength. Chiellini et al. [48] prepared a blended bioplastic product from a mixture of starch, polyvinyl alcohol (PVA), and microalgae (*Ulva armoricana*). The bioplastic showed satisfactory film-forming and mechanical characteristics. Bulota and Budtova [142] synthesized bioplastics with a tensile strength of 45 MPa by mixing algal biomass with PLA (20:80).

**Figure 2:**
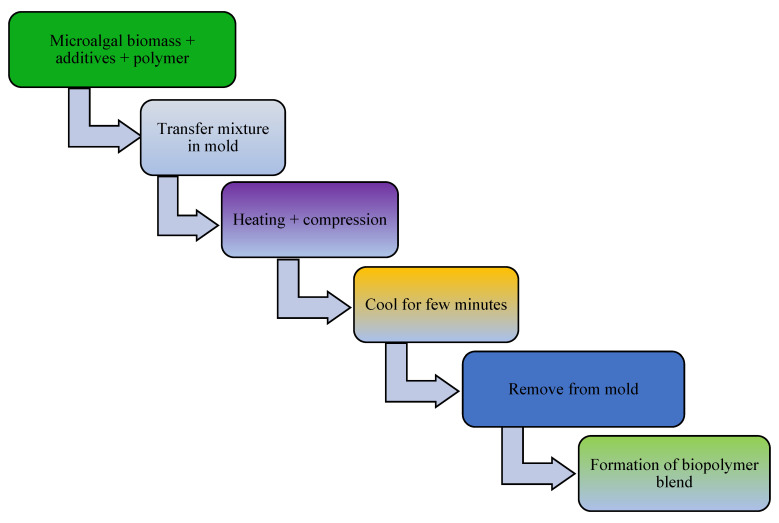
Schematic diagram showing conversion of microalgal biomass to bioplastics by the compression molding technique.

**Figure 3:**
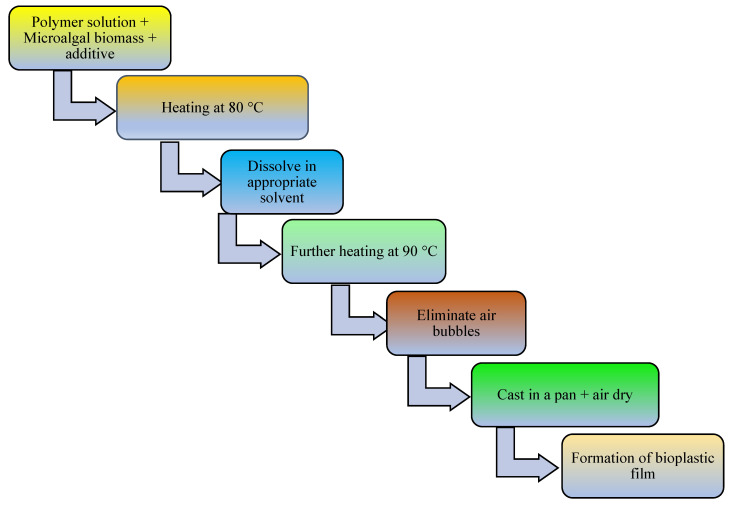
Schematic diagram showing conversion of microalgal biomass to bioplastics by the solvent casting technique.

**Figure 4:**
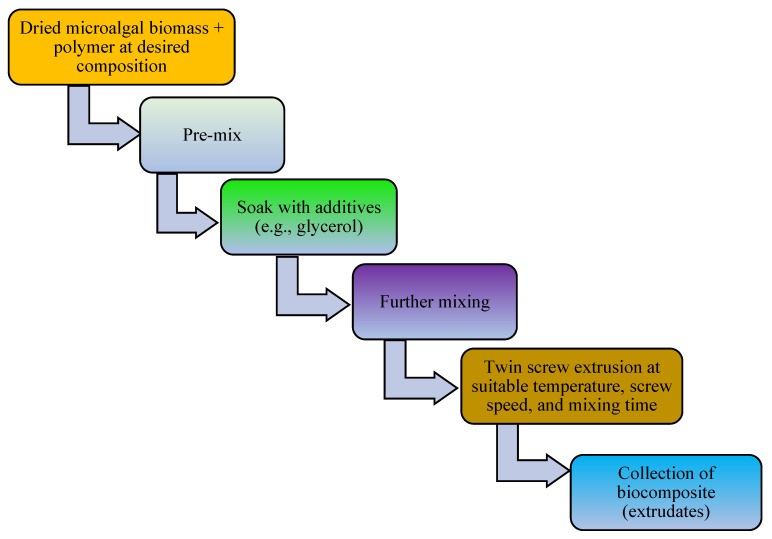
Schematic diagram elucidating conversion of microalgal biomass to bioplastics by the twin screw extrusion technique.

**Figure 5:**
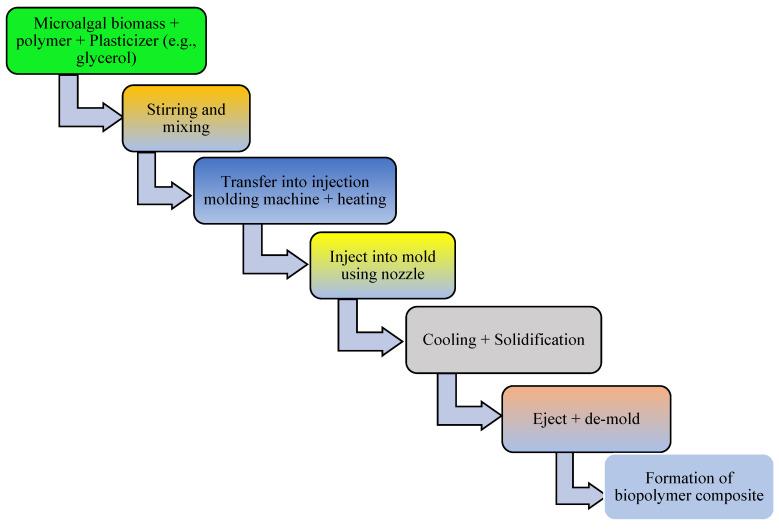
Schematic diagram depicting conversion of microalgal biomass to bioplastics by the injection molding technique.

The addition of a plasticizer (e.g., glycerol) and compatibilizer (such as maleic anhydride, diethyl succinate) to biopolymer mixtures enhances the flexibility, processability, tensile strength, thermal stability, miscibility, and elongation of the produced bioplastics [13]. For example, one study examined a blended bioplastic consisting of PE, glycerol, and *Spirulina*. The incorporation of glycerol improved the extensibility, flexibility, and tensile load of the biobased bioplastic [50]. The number of OH groups in the plasticizer, its compatibility with the biopolymer, and its concentration and type determine the success of bioplastic formation [67]. In addition, the compatibilizer homogenizes the interfacial bonding between synthetic polymers and algae-based biopolymers [151]. Other potential additives in bioplastic production include surface modifiers (such as gluten), which improve the phase morphology, performance, miscibility, and thermoplastic properties of algae-based bioplastics [13,152,153]. Thereafter, the bioplastics are molded or extruded in the presence of heat and pressure in preparation for the desired use. Microalgae-polymer blends are achieved with the aid of techniques such as compression molding, solvent casting, twin-screw extrusion, or injection molding (Figure 2, Figure 3, Figure 4 and Figure 5). For instance, in one study, a cocktail of microalgae (biofiller), glycerol, 1,4-butanediol, octanoic acid (plasticizer), and wheat gluten was transformed into bioplastic by compression molding at 40,000 kPa and 120 °C for 600 s. The obtained film demonstrated 22% elongation at break, 4.9 tensile strength, and 1 MJm^−3^ toughness [154]. Abdol and Ali [79] employed solvent casting for the preparation of bioplastic by melt-mixing biomass from three microalgal species (*Haemaatococcus pluvialis*, *Microcystis aeruginosa*, and *Chroococcus turgidus*) with glycerol, sorbitol, and gelatin, leading to the formation of PHB with a tensile strength of 1.62 MPa and elongation at break of 530%. Mathiot et al. [122] synthesized a *Chlamydomonas reinhardtii*-starch biocomposite by twin screw extrusion in the presence of 2.34% water at a melting point of 159 °C. In addition, a combination of microalgal biomass and polybutylene adipate terephthalate (PBAT) was subjected to twin extrusion at 100 rpm and 100 °C for 120 s. The resultant mixture was injection molded at 30 °C in the presence of plasticizers (urea and glycerol), leading to the formation of a biopolymer with elongation of 600% and tensile strength of 21 MPa [57].

### Cultivation of Microalgae in Wastewater for Bioplastics Production

Microalgae are cultivated in wastewater (open systems) for simultaneous treatment of waste effluent and synthesis of suitable value-added products through production of biomass [123]. The physiological characteristics of the microalgal species and the availability of nutrients, pH, temperature, and light, as well as the physiochemical properties of the wastewater, influence the efficiency of the phototrophic organisms in wastewater treatment and bioproducts synthesis [123,155,156]. The biomass can be employed for the production of biopolymers, depending on its chemical composition. The production of biopolymers and bioplastics from microalgal biomass, when grown in wastewater, ameliorates wastewater treatment costs and promotes environmental sustainability [157]. This approach is sustainable, highly flexible in terms of raw materials and products, and generates less waste [51,158,159]. However, biological or inorganic contamination of the wastewater can affect biomass composition, as well as the economic and technical viability of the system, thereby minimizing the quality and yield of desired biopolymers [123]. In this technique, cellular biomass is produced without demand for a synthetic culture medium. For instance, Lopez Rocha et al. [160] cultivated a cocktail of microalgae, including *Arthrospira platensis*, *Scenedesmus obliquus*, *Nannochloropsis gaditana*, and *Desmodesmus communis* in municipal wastewater. The consortium of biomass obtained was added to glycerol and subjected to injection molding at 140 °C for the synthesis of highly thermostable and low-water absorption bioplastic. Similarly, *Botryococcus braunii*, when grown in sewage wastewater, resulted in a PHB yield of 247 mg/L [161]. Meixner et al. [162] cultivated *Synechocystis salina* in anaerobic digestate. The experimental results showed a maximum PHB concentration of 95.4 mg/L in diluted supernatant. In addition, the hydrolysis of wastewater-derived microalgal biomass is another approach useful for bioplastics production. For example, in one study, recombinant *E. coli* was cultivated on hydrolyzed biomass [10]. Remarkably, Rahman et al. [163] supplemented recombinant *E. coli* growth media with hydrolyzed algal biomass (harvested from wastewater), resulting in the production of PHB equal to 31% of the *E. coli* dry cell weight.

## 5. Strategies for Optimization of Bioplastics Production

**Figure 6:**
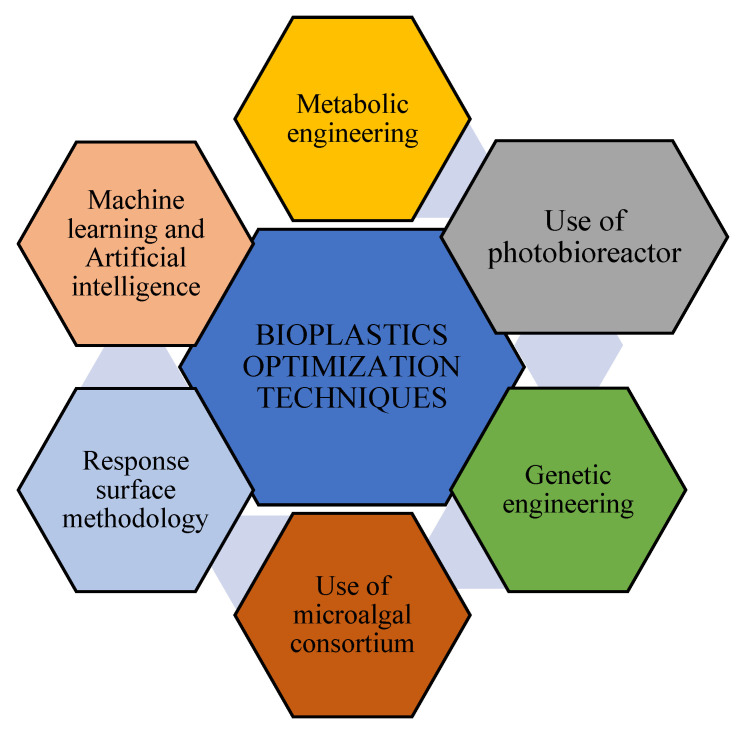
Schematic diagram showing various methods for optimization of bioplastics production by microalgae.

Different techniques are employed to enhance the microalgal synthesis of bioplastics at lower costs. The various methods used to optimize commercial bioplastics production by microalgae are illustrated in Figure 6, and some of the techniques are discussed in detail below.

### 5.1. Genetic Engineering

The upsurge in the demand for quality bioplastics necessitates the development of innovative techniques for enhanced production of bioplastics with excellent properties. Genetic engineering is a commonly utilized approach due to its potential to produce hybrid materials with required properties in response to enormous market demands [10]. Such manipulation of desired genes has been reported in microalgae such as *Nannochloropsis*, *Chlamydomonas*, *Thalassiosira*, *Phaeodactylum*, *Synechocystis*, and *Synechococcus* [67,134]. However, *Synechocystis* sp. has been widely reported for its improved PHA synthesis via genetic engineering due to its optimized growth conditions, well-studied metabolic pathways, and proper characterization [164]. In general, genetic engineering is a promising technology that is easy to carry out on unicellular and phototrophic organisms such as cyanobacteria and microalgae. However, research on genome engineering of microalgae is still in its infancy, with little success reported so far [10]. Furthermore, this technique increases the intracellular PHB accumulation to a particular level, above which it can be damaging to the cell metabolism.

### 5.2. Metabolic Engineering

Metabolic engineering, involving the genetic modification of cellular machinery, is a vital technique for the enhanced production of bioplastics. This is carried out via the manipulation of cellular processes in a particular organism by altering DNA sequences, leading to mutation, or by changing the biosynthetic pathways or genes (such as inserting a desired gene in a particular organism) of the organisms, resulting in enhanced and effective production of target compounds or metabolites [10]. In other words, microalgae and cyanobacteria can be engineered with genes encoding a particular enzyme responsible for PHB synthesis [165]. In furtherance to the genes linked to the PHB pathway, the overexpression or deletion of other genes has been shown to enhance the level of acetyl-CoA and PHB biosynthesis [165,166]. The metabolic engineering of microalgal strains refines the quality of PHAs by modifying their chemical properties, including chain length, monomer composition, and molecular weight [19]. In addition, this technique improves PHB yield and produces new PHBs by increasing precursor availability, expanding substrate utilization, modifying cell morphology, and increasing the availability of cofactors [19]. This is typical of an algal strain, *Chlamydomonas reinhardtii* cc-849, which upon transformation with p105B124 and ph105C125 vectors containing phbB and phbC genes (encoding PHB synthase) from *Ralstonia eutropha*, respectively, gave rise to a high PHB accumulation of 6 µg/g, in comparison to a wildtype strain that yielded no PHB production [167]. In one study, the insertion of PHA synthesis genes from *Ralstonia eutropha* H16 in *Phaeodactylum tricornutum* resulted in a higher PHB production of 10.6% [56]. Takahashi et al. [168] prepared recombinant *Synechococcus* sp. PCC7942 following the transformation of genes (from *Alcaligenes eutrophus*) that encoded for PHB synthesis. The results obtained showed enhanced PHB concentration under nitrogen deprivation and photoautotrophic conditions. However, altering the genetic make-up of organisms makes them a potential risk to the environment upon exposure. In addition, this technique is time-consuming with regards to selection and isolation of desired mutants, expensive, and requires sophisticated equipment [139].

### 5.3. Use of Photobioreactors

Photobioreactors are closed system reactors that allow the passage of light through their transparent walls for various biological processes [169,170]. In comparison to open-air systems (e.g., algal ponds), the cultivation of microalgae in photobioreactors is highly productive, resulting in huge volumetric cell densities, long-term culture maintenance, and minimal water evaporation [95,171]. However, this technology is faced with some drawbacks, including difficulty in cleaning the system, high energy demands, limited volume, high operational costs, and less light penetration owing to fouling of reactor walls [123,172]. In the design of a photobioreactor, efficient utilization of light by the organisms is among the crucial parameters for optimal microbial growth [173]. The quality, intensity, distribution, and sources of light are key for maximum biomass growth and PHA accumulation [174]. The light source could be natural, artificial, or both. The choice of suitable materials for optimum capturing of light is imperative in the design of a photobioreactor. Some suitable materials include glass, polyvinyl chloride (PVC), acrylic PVC, and polyethylene [175]. The use of glass walls in the construction of photobioreactors is durable and permeable with excellent mechanical strength [176]. The agitation of culture medium in photobioreactors enhances mass transfer, circumvents cell sedimentation, permits cells to receive equal light intensity, and reduces nutrient gradients [173,177]. Photobioreactors are grouped into three different categories: fixed growth biofilm systems, suspended systems, and immobilized systems. Various microalgae, such as *Chlorella vulgaris*, *Chaetomorpha maxima*, and *Haematococcus pluvialis*, have been cultivated in membrane photobioreactors, moving-bed biofilm photobioreactors, and porous-substrate photobioreactors, respectively [178,179,180]. Troschl et al. [82] employed a semi-continuous photobioreactor for the cultivation of *Synechocystis* sp. CCALA192 in the presence of carbon dioxide as a substrate. The experimental results yielded 12.5% PHB accumulation by the microalgal strain. Meixner et al. [162] cultivated *Synechocystis salina* in anaerobic digestate fractions for 40 d using a 200 L pilot-scale tubular photobioreactor. The authors reported a PHB concentration of 89 mg/L in the diluted supernatant at an illumination intensity of 9.6 W/m^2^.

### 5.4. Use of Machine Learning and Artificial Intelligence

The use of machine learning (ML) and artificial intelligence (AI) has revolutionized the development of microalgae-derived bioplastics for a circular economy and sustainable future. In addition, these technologies provide real-time monitoring, remote control, and predictive modelling of bioplastics production. The incorporation of these technologies paves ways for improved sustainability and efficiency in bioplastic production [180]. Several studies have reported on the identification, classification, and cultivation of microalgae for bioplastic synthesis using ML algorithms and AI-based systems [181,182,183]. ML can effectively optimize microalgal strains by predicting and selecting strains with optimal bioplastic aptitudes. In addition, ML is employed to optimize bioplastic production processes by maximizing yields and lessening energy intake and waste generation [184,185].

### 5.5. Response Surface Methodology

Response surface methodology (RSM) is a combination of mathematical and statistical techniques, used for the design of experiments, modelling, and the selection of optimum conditions or parameters that influence a particular response [186]. In general, RSM is employed for the optimization of bioprocess parameters for enhanced microbial growth and secretion of desired metabolites (products) [187]. In addition, this technique has been used to identify effective variables, study interactions, and quantify relationships between responses in a limited number of experiments [188]. Experimental designs, including central composite design (CCD), Box–Behnken design (BBD), and Doehlert design, are commonly used in RSM. Response surface methodology has been employed for the optimization of bioplastics production by various researchers [189,190,191]. For instance, Kavitha et al. [161] optimized temperature, pH, and substrate for enhanced PHB production by *Botryococcus braunii* using RSM. The authors recorded a maximum yield of 247 ± 0.42 mg/L, found to be in close agreement with the predicted yield of 246 ± 0.32 mg/L at optimum sewage wastewater concentration (substrate) of 60%, pH 7.5, and 40 °C. Yashavanth and Maiti [192] recorded a 2.72-fold enhancement in PHB production by *Chlorogloea fritschii* TISTR 8527 following optimization of NaNO_3_, K_2_HPO_4_, TRACE X, Na_2_EDTA, and MgSO_4_.7H_2_O, using CCD of RSM.

## 6. Life Cycle Assessment of Bioplastics Production

Life cycle assessment offers a quantitative, qualitative, and detailed understanding of the environmental impacts of bioplastics by appraising their entire life cycle based on indicators such as global warming potential, human toxicity, eutrophication, ecotoxicity, and acidification potential, amongst others [13]. It is employed to measure the sustainability of a product or process [72]. It involves various analytical methods, including cradle-to-grave (acquisition, production processes, use, and end-of-life), cradle-to-gate (acquisition of resources and production processes), and gate-to-grave (use and end-of-life stage) [67]. Bioplastics minimize greenhouse gas emissions and eliminate hazardous production steps. For instance, the replacement of polyethylene terephthalate (PET) bottles with PLA bottles by Atiwesh et al. [193] resulted in a 20% reduction in greenhouse gas emissions, while also saving two-thirds of the energy consumed in the fabrication of synthetic plastics. Similarly, the synthesis of bio-PE was found to generate greenhouse gas emissions of about 0.75 kgCO_2eq_/kg PE, 140% less than the production of fossil-based PE, with consequential savings of nearly 65% on the consumption of non-renewable energy [194].

The life cycle of various bioplastics was examined by Álvarez-Chávez et al. [195] based on their environmental and health hazards, such as exposure to toxic additives and solvents during production, energy and water use efficiency, release of toxic by-products, and the genetic manipulation of feedstock. The authors reported sustainable improvements for PHA and PLA biobased polymers when compared to petrochemical polymers. Based on a cradle-to-gate approach, Harding et al. [196] reported a useful PHB biosynthesis method, found to be sustainable and eco-friendly owing to substantial decreases in toxicity levels, acidification, abiotic depletion values, and ozone layer depletion levels in comparison to polypropylene production. In addition, Beckstrom [197] assessed the intensity of greenhouse gases during the cultivation of microalgae for bioplastic synthesis in different systems. A cyclic flow photobioreactor demonstrated robust impact values in contrast to open raceway ponds and other combined systems. Rueda et al. [72] carried out a life cycle assessment for PHB production by *Synechocystis* sp. R2020 to evaluate the sustainability of the bioplastic synthesis. Their findings showed that increasing the PHB content in the microalgal biomass severely reduced (67–75%) the environmental impacts of the production process. The environmental impacts were caused by using chemicals (such as chloroform for PHB purification) and construction materials. Similarly, Araujo et al. [198] reported that greener synthesis of cellulose-based polymer (cellulose acetate) is sustainable with lower environmental impacts in comparison to traditional processing approaches.

The end-of-life of bioplastics is considered a vital aspect of life cycle assessment. It involves the use of different techniques for the effective management and disposal of bioplastics. These include recycling, landfilling, incineration, anaerobic digestion, and composting. Mechanical recycling is considered excellent for the management of bioplastics, owing to its inexpensiveness, lower carbon footprint, and inability to emit unpleasant gases [67]. For instance, Rosenboom et al. [199] reported a lesser CO_2_ generation (0.62 kg) during mechanical recycling of PLA when compared to anaerobic digestion, composting, incineration, and landfilling.

## 7. Applications of Bioplastics

### 7.1. Agricultural Applications

**Figure 7:**
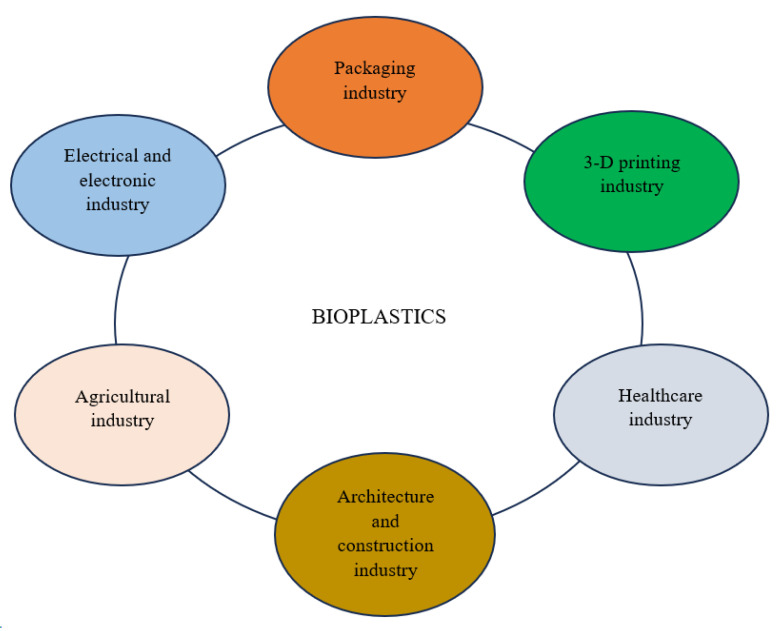
Schematic diagram illustrating different applications of bioplastics.

Bioplastics are employed in agriculture as mulch films, grow bags, tunnels, pots, seedling trays, pesticide containers, and farm nets due to their biodegradability, durability, protective properties, water resistance, compostability, etc. [86,200,201] (Figure 7; Table 2). Bioplastic mulch contributes to 40% of the mulch used in agriculture. It is prepared using materials such as starch, cellulose, PHA, and PLA, and can be easily degraded by soil microbes, thereby enhancing soil fertility and crop yield [202]. Furthermore, bioplastic mulch maintains soil conformation, retains soil moisture content, and protects crops from weeds growth, insects, and birds [203]. In addition, PHA can be used as a support material (carrier) for seed encapsulation, crop protection films, and insecticides [32]. Due to their elasticity and strength, bioplastics are also employed in the packaging of farm produce. Furthermore, bioplastics speed up the degradation of other polymers in the soil, enhancing their suitability for use in nursery bags or pots [86,204]. Agricultural nets are often made from PHB or bioplastics blended with PLA because of their high tensile strength [205].

### 7.2. Industrial Applications

Bioplastics are employed in an enormous range of applications in the electrical and electronic, packaging, architecture, and construction industries, among many others (Figure 7; Table 2). In the electrical and electronic industries, these biopolymers are employed as bioplastics conductors for the design of diodes, batteries, fuel cells, and electrochromic devices [211]. Bioplastics are also used as casings during assemblage of devices, including mobile phones, computer accessories, and speakers, among others, as well as membranes for sound-transmitting and amplifying devices [210,212]. In addition, bioplastics strengthened with carbon nanotubes and cellulose nanofiber are used in sensors, photovoltaic cells, and advanced electronics technologies [208]. Furthermore, some 3-D printing filaments consist of graphene-supported PLA, which provides a faster cooling rate owing to its biodegradability, excellent thermal conductivity, and minimal deformation [209].

Bioplastics are commonly used as films, wraps, and bottles for beverages and dairy products, containers, dishes, and takeaway bags in packaging industries. However, due to their low permeability, these biopolymers create great challenges in the packaging industry [215]. As a result, bioplastics for these applications are often supported with additional materials [219]. The use of reinforced bioplastics extends the lifespan of fresh fruits and food products when used for packaging [21]. For instance, cellulose-based films reinforced with clay and PBAT supported with thermoplastic starch bioplastic films were shown to demonstrate robust thermal stability, gas permeability, and antimicrobial properties when used for food packaging [22,215]. In the construction industry, reinforced bioplastics are used in doors, window frames, and construction textiles, and as stabilizers for earthen construction materials, as well as insulation for partitions and walls in temporary constructions [217,218].

### 7.3. Medical Applications

Bioplastics are used in a wide variety of biomedical applications due to their biodegradability, biocompatibility, porosity, and non-toxicity (Figure 7; Table 2). These include the development of therapeutic devices (such as 3-D scaffolds and implants for tissue engineering) and as vehicles for controlled drug release [85]. PHAs are used for cancer detection, drug delivery agents, post-surgical ulcer therapy, bone tissue engineering, heart valve implants, and wound-healing dressings [19,214]. In addition, PHBHHx and hydroxyapatite blended with PHB have been shown to improve bone tissue growth and cell division, respectively [220]. Lignin-reinforced bioplastics with high antioxidant activity protect humans from skin radiation, oxidative stress, and facilitate regeneration of cartilage tissue [213]. In addition, polyhydroxyoctanoate and polyethylene glycol copolymer nanoparticles have been employed for the delivery of paclitaxel, an anticancer drug in mice, resulting in reduction in colon carcinoma [221]. Furthermore, biodegradable polymers are utilized as scaffolds for in vitro cell cultivation and in vivo implants. For instance, Liu et al. [222] discovered that a lecithin modified PLA-PU composite permitted the sustainable growth of hepatocytes (HePG2 cells) in comparison to conventional cultivation in culture plates. The implantation of PHB film patches into mice with cranial defects was carried out by Gredes et al. [223]. The patches promoted bone formation with enhanced blood vessel development. However, despite the enormous range of applications of bioplastics in biomedicine, these biopolymers possess some limitations, including the need to undergo sterilization processes, thereby increasing the degradation rate, and reducing the molecular weight of the bioplastics [86]. In addition, the identification of suitable polymers for appropriate medical applications is determined by the polymer chemistry, performance, processing, and device design [85].

## 8. Some Challenges Confronting Large-Scale Bioplastic Production

The production of bioplastics from microalgae is promising and eco-friendly. However, this technique is faced with some limitations, which make its commercialization unachievable. These include the following:

(i) Bioplastic production is associated with high costs relating to production and downstream processing. For this challenge to be ameliorated and make bioplastic synthesis economically viable, the choice of raw materials used in the production process is vital since substrate costs account for less than 70% of the overall production costs. Therefore, the use of less expensive and readily available raw materials (e.g., molasses) will pave the way for the commercial synthesis of bioplastics. In addition, the use of economical, efficient, and sustainable methods for the optimal recovery of biopolymers is imperative for low-cost bioplastics production [19].
(ii) Irregularities in bioplastic properties and low substrate-to-product conversion ratios are critical bottlenecks affecting large-scale bioplastics production.
(iii) The proper identification of microalgae capable of producing biopolymers for the synthesis of bioplastics with varying properties is a great challenge in the bioplastics industry [55].
(iv) The selection of suitable polymers from microalgae is also recognized as a challenge in the production of bioplastics with excellent tensile strength. This is based on several criteria, including biodegradability, brittleness, moisture content, and molecular weight [224].
(v) There exists a lack of awareness among consumers regarding the usefulness of bioplastics. This can be mitigated by increasing marketing strategies, coupled with cost-effective and biodegradable production processes that do not generate greenhouse gas emissions. Educating the public on the environmental and health benefits of bioplastics will lead to increased acceptance and demand.
(vi) The indiscriminate disposal of bioplastics in the environment causes severe hazards. This can be alleviated by adequate waste management practices using methods such as landfilling, anaerobic digestion, composting, and incineration. However, composting is the most preferred technique because it allows rapid degradation of the bioplastics within a short period of time [139].

## 9. Concluding Remarks and Future Perspectives

The use of microalgae as a bio-factory for the synthesis of bioplastics has attracted significant attention due to the ability of these photoautotrophic organisms to grow rapidly with less nutrients. Microalgae produce a variety of biopolymers, including PHA, PLA, PU, cellulose-based polymers, starch-based polymers, and protein-based polymers, when cultivated under different conditions. These polymers have great potential and interesting properties, including biodegradability, biocompatibility, and non-toxicity. Techniques such as genetic engineering, metabolic engineering, the use of photobioreactors, artificial intelligence, and machine learning are currently being employed for large-scale and inexpensive production of bioplastics from microalgae for applications in the agriculture, healthcare, packaging, electrical and electronic, and construction industries. The recommendations for future directions include the following:

(i) Further studies on microalgae biorefinery involving the use of genetic engineering and metabolic engineering as vital tools for enhanced biomass production and purity should be carried out to achieve high quality novel bioplastics at lower costs.
(ii) The development of energy efficient and cost-effective photobioreactors will provide controlled culture conditions for enhanced microalgae biomass yields for bioplastics synthesis.
(iii) Further research on bioprospecting for novel hyperactive microalgal strains and the application of consortium of microalgae is crucial for industrial scale production of bioplastics with less additives, thus promoting circular economy for a sustainable future.
(iv) A proper understanding of the mechanisms of bioplastics accumulation in microalgae is imperative to pave the way for more research opportunities.
(v) The use of different compatible natural reinforcing agents should be the focal point of future research for the synthesis of bioplastics with greater tensile strength and robust thermal stability.

## Figures and Tables

**Table 1:** Bioplastics yields and cultivation conditions from different microalgal species.

Microalgae Species	Culture Condition	Yield	Reference
*Synechococcus elongatus* UAM-C/SO_3_	Nitrogen limitation	29.4% PHB	[143]
*Oscillatoria okeni* TISTR 8549	Nitrogen limitation, acetate supplementation, dark condition, heterotrophic, and 6.5% 3-hydroxyvalerate	42% P(3HB-co-3HV)	[144]
*Scenedesmus* sp.	0.021 mM Fe, 0.5 g/L salinity, and 17.6 mM Nitrogen	29.92% PHB	[68]
*Botryococcus braunii*	60% sewage water as culture medium	247 mg/L PHB	[144]
*Aulosira fertilissima* CCC 444	Phosphorus deprivation, 0.4% valerate, and 0.5% fructose	77% P(3HB-co-3HV)	[145]
*Aulosira fertilissima* CCC 444	0.28% acetate, 0.26% citrate, incubation period 5 d, and 5.58 mg/L K_2_HPO_4_	85% PHB	[145]
*Nostoc muscorum*	Dark condition, incubation period 7 d, and 0.2% acetate	35% P(3HB)	[53]
*Calothrix scytonemicola* TISTR 8095	Nitrogen limitation, CO_2_ as carbon source	25.4% P(3HB)	[77]
*Chlorella fusca* LEB 111	D-xylose addition	17.4% PHB	[146]
*Synechocystis* sp. UNIWG	Nitrogen deficiency	14% PHB	[147]
*Spirulina platensis*	Acetate and CO_2_ as carbon source	10% P(3HB)	[148]
*Chlamydomonas reinhardtii*	Nitrogen limitation, D-xylose addition	206 mg/L PHB	[146]
*Nostoc muscorum*	pH 8.5, 0.4% propionate, incubation period 14 d, and 0.2% acetate	28.2% P(3HB)	[54]
*Chlorella* sp.	pH 7.0, 30 °C, sunlight, 0.2% sodium bicarbonate	80% PHB	[80]
*Desmodesmus communis*	3 cultivation, batch, intracellular, low light, phosphorus-free medium, and 1 g/L sodium acetate	32.1% PHB	[149]
*Chlorella sorokiniana* SVMIICT8	pH 7.0, sodium acetate as carbon source, light-dark period, and aeration	29.5% PHB	[150]

**Table 2:** Applications of bioplastics in different industries.

Industry	Product or Application	Reference
Agriculture	Seedling trays, mulch film, farm nets, pots, and nursery bags	[19,21,96,206,207]
Electrical and electronic	Diodes, batteries, fuel cells, electrochromic devices, casings (electronic devices), membranes (sound-transmitting and amplifying devices), sensors, photovoltaic cells, and 3-D printing filaments	[208,209,210,211,212]
Medical	Cancer detection, tissue engineering, drug delivery agents, post-surgical therapy, implants, wound healing dressings, antioxidant activity, production of biomedical devices	[19,23,85,89,213,214]
Packaging	Films, wraps, containers, bottles, takeaway bags, and dishes	[22,215,216]
Construction	Doors, construction materials, windows, frames, insulation, and walls	[217,218]
